# Exploring socioeconomic inequalities in the use of medicinal herbs among Iranian households: evidence from a national cross-sectional survey

**DOI:** 10.1186/s12906-020-03131-y

**Published:** 2020-11-09

**Authors:** Satar Rezaei, Sina Ahmadi, Amjad Mohamadi-Bolbanabad, Ahmad Khanijahani

**Affiliations:** 1 Research Center for Environmental Determinants of Health, Health Institute, Kermanshah University of Medical Sciences, Kermanshah, Iran; 2 Department of Social Welfare Management, University of Social Welfare and Rehabilitation Sciences, Tehran, Iran; 3 Social Determinants of Health Research Center, Research Institute for Health Development, Kurdistan University of Medical Sciences, Sanandaj, Iran; 4 Department of Health Administration and Public Health, Rangos School of Health Sciences, Duquesne University, 600 Forbes Avenue, Pittsburgh, PA 15282 USA

**Keywords:** Medicinal herbs, Complementary and alternative medicine, Inequalities, Socioeconomic status, Iran

## Abstract

**Background:**

Association between socioeconomic status and medicinal herbs (MH) are rarely documented in Iran. Our goal was to measure and decompose socioeconomic inequalities in MH use among Iranian households.

**Methods:**

The data used in this cross-sectional study were extracted from the 2018 Household Income and Expenditure Survey (HIES) (*N* = 38,859). Data on MH use, age, gender, and education status of the head of household; a constructed wealth index of household (as a proxy for household’s socioeconomic status); and place of residence (urban or rural) were obtained from the survey. Publicly available province-level data on Human Development Index (HDI) were obtained from the Institute for Management Research at Radbound University. We used the concentration curve and the normalized concentration index (*C*_*n*_) to measure the magnitude of socioeconomic inequalities in MH among Iranian households. The *C*_*n*_ was decomposed to identify the main determinants of socioeconomic inequalities in MH in Iran.

**Results:**

The overall prevalence of MH use among Iranian households was 4.7% (95% confidence interval [CI]: 4.5 to 4.9%) in the last month before data collection. The *C*_*n*_ for MH use for the whole of samples was 0.1519; 95% CI = 0.1254 to 0.1784; suggesting a higher concentration of MH use among the households with high socioeconomic level. The decomposition analysis indicated that the main contributing factors to the concentration of MH use were the economic status of households, development status of the province, and education level of the household head.

**Conclusions:**

This study demonstrated that MH use is more concentrated among socioeconomically advantaged households in Iran and its provinces. This finding might contrast with the widespread belief that wealthy and socioeconomically advantaged populations, compared to low SES groups, tend to seek disproportionately more modern medical treatments and medications than MH. Understanding the factors affecting MH use, socioeconomic inequality in use of MH and its determinants provide an opportunity for health policymakers to design effective evidence-based interventions among providers and consumers of MH.

## Background

The use of medicinal herbs (MH) is increasing both in developing and developed countries [1, 2]. As per World Health Organization (WHO) definition, traditional medicine is “the sum total of the knowledge, skill, and practices based on the theories, beliefs, and experiences indigenous to different cultures, whether explicable or not, used in the maintenance of health as well as in the prevention, diagnosis, improvement or treatment of physical and mental illness” [2]. Besides, the term complementary medicine refers to “a broad set of health care practices that are not part of that country’s own tradition or conventional medicine and are not fully integrated into the dominant health-care system” [2]. MH is related to a range of medical interventions that are primarily used outside the formal healthcare setting. For millions of people in most countries, especially developing countries, MH is an important part of health systems and is considered as one of the main sources of health services and, in some cases, are the only sources for care. MH is usually close to home, accessible, and affordable [3]. The concept of MH has increasingly gained interest among herbalists and the scientific community worldwide. MH is defined by the World Health Organization (WHO) as “herbs, herbal materials, herbal preparations and finished herbal products, that contain as active ingredients parts of plants, or plant materials, or combinations thereof” [2, 4].

Previous studies report that a high percentage of people worldwide use medicinal to treat various health conditions. Its prevalence ranges from 5 to 90% in different geographical, social, and cultural settings [5–11]. A systematic review study found that the prevalence of complementary and alternative medicine use varied between 0.3 to 86% among European Union countries [12]. Stanifer and colleagues (2015) also found that traditional medicine use has increased among all the groups of people, especially those with higher educational attainment and professional occupations, and across all ages in urban and rural residents [13].

Because of the historical background, geographical location, and various climatic conditions, Iran is a habitat of many medicinal plants species. Traditional medicine has always been practiced despite advances in modern medicine [14]. There are more than 7500 plant species in Iran, and about 1800 of those species are considered medicinal [15]. In recent years, MH has become increasingly popular among Iranians. Hashempur et al. [16] investigated the prevalence of use of complementary and alternative medicine (CAM) in Iranian patients with diabetes mellitus in the Shiraz province. Of the 239 patients included in their study, 75.3% used at least one type of CAM over the last year.

Existing literature highlights that many factors (e.g., education, income, ethnicity, accessibility, and affordability) affected MH use in different countries [17–19]. In the case of Iran, most of the studies on MH use and its main determinants have been focused on specific groups such as pregnant women and those with special health conditions like hypertension, diabetes, HIV, and mental health disorders [6, 11, 18, 20–22]. The prevalence of MH use and its determinants among the general population at the household level is rarely documented. To fill this gap in the current literature, this study aimed to measure and decomposes socioeconomic inequalities in MH use among Iranian households at the national and sub-national levels.

## Methods

### Study setting

Iran is a middle-income country located in the Eastern Mediterranean Region (EMR) with an area of 1648 million square kilometers. According to the 2016 census data, Iran's population was approximately 80 million people living in 31 provinces [23]. In the healthcare system in Iran, health care is provided by three sectors, including public, private, and not-for-profit. While the public sector plays a key role in the provision of all three levels of healthcare (i.e., primary, secondary, and tertiary), the private sector mainly provides secondary and tertiary healthcare in urban areas. Non-governmental organizations (NGOs) are active in providing health services for chronic patients, diabetes patients and severe patients, such as cancer patients [24]. MH in Iran are easily available at Atari (an herb store where provide CAM to Iranian people), and the people do not need to visit medical doctors to get prescription.

### Data and variables

The data used in this study was extracted from the 2018 Household Income and Expenditure Survey (HIES) conducted by the Iranian Statistical Center [25]. This is a nationwide cross-sectional survey consisting of face-to-face interviews with household heads. Household is the unit of data collection, report, and analysis in the HIES. Households were selected using three-stage cluster sampling. In the first stage, the census areas are classified and selected. The urban and rural blocks are selected in the second stage. In the final stage, households in the sample were selected. Information on sociodemographic characteristics of household (e.g., the age of household head, the gender of household head, and education status of household head); household healthcare utilization (e.g., MH); and household assets, income, and expenditure over the past month were collected in the survey. In 2018, data were collected from 38,859 rural and urban households.

The dichotomous dependent variable of MH use was constructed using the respondents’ answer to if the household used any medicinal herbs (e.g., borage, purgative manna, the manna of Hedysarum, Cichorium, etc.) over the past month. Based on the availability of data in the HIES and previous studies [17–19], we used the gender of household head, age of household head, education status of household head, a constructed wealth index of households (as a proxy for households’ socioeconomic status), residential area (urban/rural), and development stats of province based on their HDI score (low, middle, and high) as the determinants of MH use in households. Province-level data on HDI was accessed from the Institute for Management Research at Radboud University [26]. Principal Component Analysis (PCA) was used to construct a household wealth index [27]. We included the housing characteristics (e.g., rooms per capita, type of house ownership, and house size per square meter) and durable assets of households (e.g., car, TV color, internet, computer/laptop, cell phone, freezer, dishwasher, microwave, vacuum cleaner, motorcycle, and bicycle) in the PCA procedure. Households were classified into five socioeconomic status (SES) groups ranging from the poorest (first quintile) to the richest (fifth quintile) according to the wealth score.

### Data analysis

#### Measuring socioeconomic inequalities in the use of medicinal herbs

We used Concentration index (C) and Concentration curve [28] to examine and quantify the socioeconomic-related inequalities in MH use among households in Iran and its provinces. In the Concentration curve, the cumulative proportion of households ranked by SES and the cumulative proportion of MH use are plotted on x and y axes, respectively. If the concentration curve lies above the 45-degree line. This suggests that MH use is concentrated more among poor households and vice versa. The C takes a value between − 1 and + 1, with the value of zero meaning no socioeconomic-related inequalities. A negative sign of the C indicates that MH use is more concentrated among households with low SES and vice versa. AS the MH use is a binary (is either 0 or 1) measure, and the estimated C is not between − 1 and + 1. Thus, as suggested by Wagstaff [29], we normalized the Concentration index (C_n_) by dividing it by $\frac{1}{1-\upmu}$, where μ is the mean of the use of MH.

#### Decomposing socioeconomic inequalities in the use of medicinal herbs

We used the decomposition methods to identify the main factors associated with the observed socioeconomic-related inequalities in use of MH. In the decomposition analysis, a set of k explanatory factors, x_k_, were regressed on the outcome variable, y (here use of MH) in a linear regression model as follows:

1 $$ \mathrm{y}=\upalpha +\sum \limits_{\mathrm{k}}{\upbeta}_{\mathrm{k}}\ {\mathrm{x}}_{\mathrm{k}}+\upvarepsilon . $$

The C for MH use can be decomposed using the followng equation [30]:

2 $$ \mathrm{C}=\sum \limits_{\mathrm{k}}\left(\frac{\upbeta_{\mathrm{k}}{\overline{\mathrm{x}}}_{\mathrm{k}}}{\upmu}\right){\mathrm{C}}_{\mathrm{k}}+\frac{\mathrm{G}{\mathrm{C}}_{\upvarepsilon}}{\upmu}. $$

Where ${\overline{\mathrm{x}}}_{\mathrm{k}}$ is the mean of x_k_, μ presents the mean of use of MH , C_k_ shows the C for x_k_, and $\left(\frac{\upbeta_{\mathrm{k}}{\overline{\mathrm{x}}}_{\mathrm{k}}}{\upmu}\right){\mathrm{C}}_{\mathrm{k}}$ is the elasticity of MH use with respect to the x_k_. A positive (negative) elasticity for a factor shows that the probability of MH use among households increasing (decreasing) when the value of that factor also increases (decreases). ${\sum}_{\mathrm{k}}\left(\frac{\upbeta_{\mathrm{k}}{\overline{\mathrm{x}}}_{\mathrm{k}}}{\upmu}\right){\mathrm{C}}_{\mathrm{k}}$ denotes the contribution of explanatory factors, x_k_, to the overall C for the use of MH. The last term, $\frac{\mathrm{G}{\mathrm{C}}_{\upvarepsilon}}{\upmu}$, is the residuals part and indicates the portion of the C for use of MH that cannot be explained by the explanatory variables included in the analysis.

Wagstaff et al. [29] showed that the Cn can be decomposed to its determinants using the following formula:

3 $$ {\mathrm{C}}_{\mathrm{n}}=\frac{\mathrm{C}}{1-\upmu}=\frac{\sum_{\mathrm{k}}\left(\frac{\upbeta_{\mathrm{k}}{\overline{\mathrm{x}}}_{\mathrm{k}}}{\upmu}\right){\mathrm{C}}_{\mathrm{k}}}{1-\upmu}+\frac{\frac{\mathrm{A}{\mathrm{C}}_{\upvarepsilon}}{\upmu}}{1-\upmu} $$

A negative absolute contribution for each factor to the C_n_ shows that the socioeconomic distribution of the respective factor and its association with MH use leads to lower MH use among the poorest households. To calculate the relative contribution of each factor, we divided the absolute contribution of that factor by the C_n_ and then multiply by 100. As MH use is a binary variable, we used the non-linear logit regression to obtain the marginal effect of determinants. All the analyses were performed using Stata Version 14. To ensure proper point and variance estimations, survey design weights and parameters were incorporated in the analyses. *P*-values less than 0.05 considered statistically significant. The geographical map was produced by ArcGIS software Version 10.6.1.

## Results

### Descriptive statistics

**Fig. 1:**
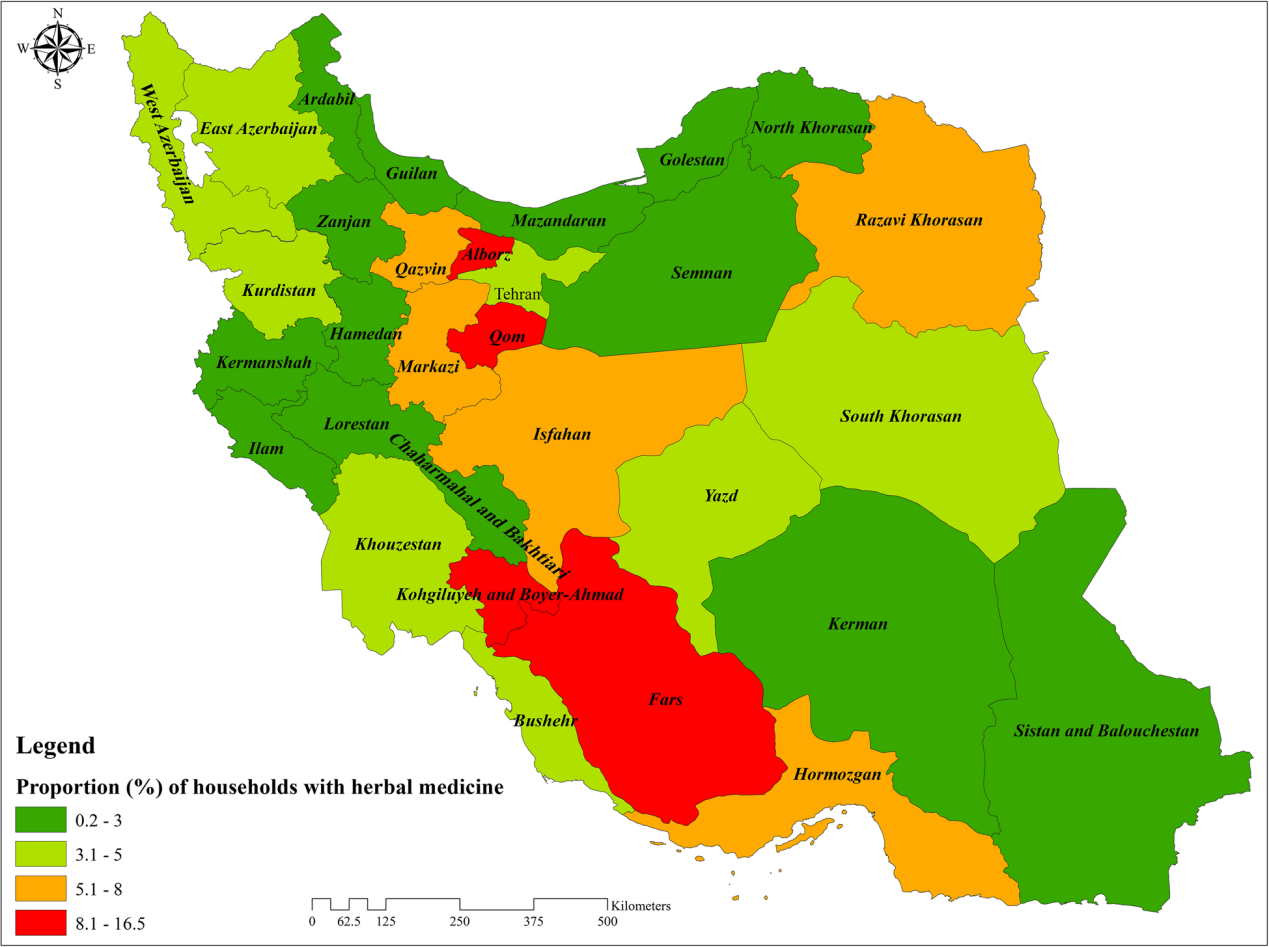
Proportion of households with use of medicinal herbs over the last month across provinces in Iran, 2018 (developed by the authors using ArcGIS Desktop version 10.6.1)

The descriptive characteristics of the 38,859 households included in this study are presented in Table 1. Of these, 86.7% (*n* = 33,752) of household heads were male. The mean age of the household heads was 50.4 years (standard deviation [SD] = 15.6). 76% (*n* = 29,524) of household heads were literate, and 88.4% (*n* = 34,370) of the study population had health insurance coverage. The overall prevalence of MH use over the past month among the households was 4.7% (95% confidence interval [CI]: 4.5 to 4.9%). The prevalence of MH use was 2.7% (95% CI: 2.4 to 3.1%) and 6.1% (95% CI: 5.6 to 6.7%) among the poorest and richest households, respectively. This figure for urban and rural households was 5.3% (95% CI: 5.0 to 5.6%) and 4.0% (95% CI: 3.8 to 4.4%), respectively. The prevalence of MH use among households across 31 provinces of Iran is demonstrated in Fig. 1. There are disparities in MH use across the provinces of Iran. The prevalence of MH usage in Lorestan and Fars provinces were 0.2 and 16.5%, respectively, representing the lowest and highest prevalence of MH use.

**Table 1:** Characteristics of the households included in the study (*N* = 38,859)

Variables	n (%) (N = 38,859)	Used Herbal Medicine n (%) (*N* = 1829)	Did not use Herbal Medicine n (%) (*N* = 37,030)
Demographic variables			
*Sex of household head*			
Male	33,752 (86.9)	1619 (4.8)	32,133 (95.2)
Female	5107 (13.1)	210 (4.1)	4897 (95.9)
*Age of household head*			
15–45	16,981 (43.7)	802 (4.7)	16,179 (95.3)
46–65	14,731 (37.9)	715 (4.8)	14,016 (95.2)
66 and above	7147 (18.4)	312 (4.4)	6835 (95.6)
Socioeconomic variables			
*Education status of household head*			
Illiterate	9335 (24.0)	353 (3.8)	8982 (96.2)
Primary school	10,808 (27.8)	459 (4.3)	1349 (95.7)
Secondary school	6965 (17.9)	325 (4.7)	6640 (95.3)
High school	7705 (19.8)	420 (5.4)	7285 (94.6)
Academic degree	4046 (10.4)	272 (6.7)	3774 (93.3)
*Wealth index of households*			
Poorest	7730 (19.9)	211 (2.7)	7519 (97.3)
Poor	7775 (20.0)	318 (4.1)	7457 (95.9)
Middle	7779 (20.0)	371 (4.8)	7408 (95.2)
Rich	7785 (20.0)	450 (5.8)	7335 (94.2)
Richest	7790 (20.1)	479 (6.2)	7311 (93.8)
Ecological variables			
*Geographical area*			
Urban	20,313 (52.3)	1077 (5.3)	19,236 (94.7)
Rural	18,546 (47.7)	752 (4.1)	17,794 (95.9)
*Province category based on HDI*			
Low	14,050 (36.2)	407 (2.9)	13,643 (97.1)
Middle	12,716 (32.7)	613 (4.8)	12,103 (95.2)
High	12,093 (31.1)	809 (6.7)	11,284 (93.3)

HDI is the human development index

#### Multivariate logistic regression analysis

The results of logistic regression to identify the main factors affecting MH among households in Iran are reported in Table 2. Rural households are less likely to use MH than their urban counterparts. Additionally, odds ratios greater than one indicates that, compared to the reference groups, households with higher socioeconomic status and provinces with higher HDI were more likely to use MH.

**Table 2:** Results of bivariate and multivariate logistic regression

Variables	Crude OR (95% CI)	Adjusted OR (95% CI)
Demographic variables		
*Sex of household head*		
Male (ref.)	1	1
Female	0.89 (0.72 to 1.10)	1.05 (0.83 to 1.32)
*Age of household head*		
15–45 (ref.)	1	1
46–65	1.00 (0.85 to 1.18)	0.96 (0.80 to 1.15)
66 and above	0.90 (0.73 to 1.10)	0.98 (0.78 to 1.24)
Socioeconomic variables		
*Education status of household head*		
Illiterate (ref.)	1	1
Primary school	1.30 (1.04 to 1.63)	1.12 (0.87 to 1.44)
Secondary school	1.32 (1.06 to 1.65)	1.05 (0.81 to 1.37)
High school	1.80 (1.44 to 2.25)	1.30 (0.98 to 1.72)
Academic degree	1.84 (1.43 to 2.38)	1.27 (0.91 to 1.77)
*Wealth index of households*		
Poorest (ref.)	1	1
Poor	1.48 (1.12 to 1.96)	1.31 (0.98 to 1.75)
Middle	1.83 (1.39 to 2.43)	1.52 (1.13 to 2.04)
Rich	2.04 (1.55 to 2.68)	1.60 (1.18 to 2.17)
Richest	2.15 (1.64 to 2.84)	1.60 (1.16 to 2.20)
Ecological variables		
*Geographical area*		
Urban (ref.)	1	1
Rural	0.59 (0.50 to 0.69)	0.73 (0.63 to 0.87)
*Province category based on HDI*		
Low (ref.)	1	1
Middle	1.33 (1.08 to 1.63)	1.23 (1.02 to 1.51)
High	2.19 (1.78 to 2.68)	1.83 (1.48 to 2.27)

OR is odds ratio and HDI is the human development index

Crude OR represents the odds ratio in a bivariate model with a single predictor

Adjusted OR represents odds ratios in the multivariate model with all predictors entered simultaneously into the model

**Fig. 2:**
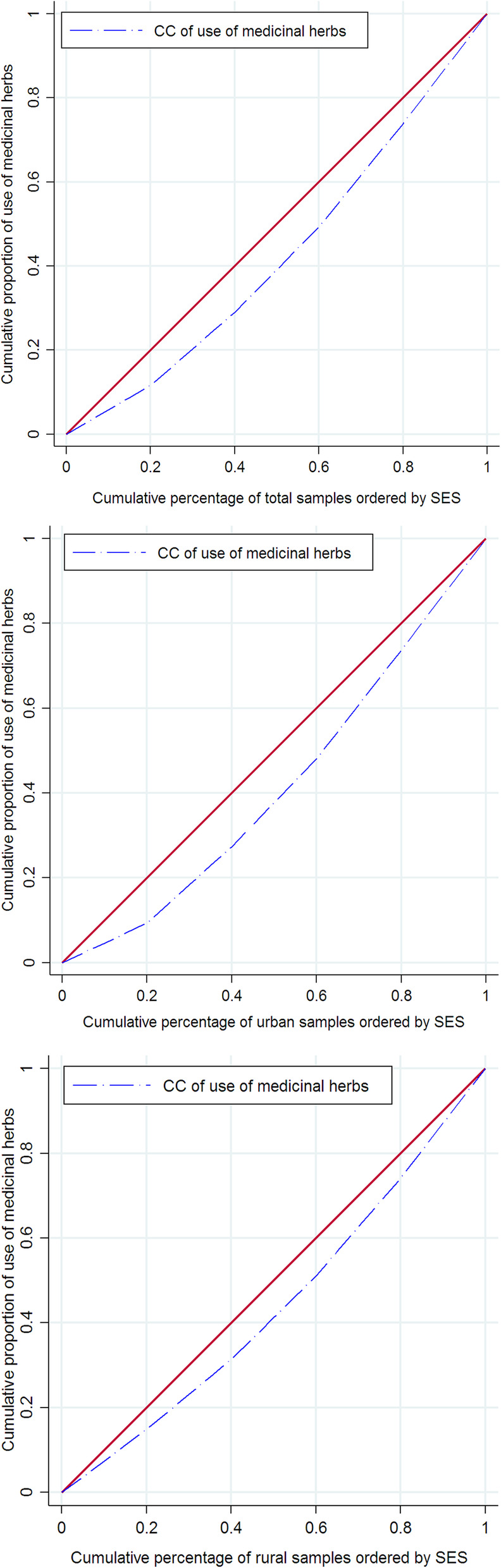
The Concentration curve for herbal medicine usage for whole sample, rural and urban areas, 2018

Table 3 shows the *C*_*n*_ for MH use among households for the whole of samples, rural and urban areas. The positive value of the *C*_*n*_ suggested that MH use is more prevalent among better-off households (*C*_*n*_ = 0.1519; 95% CI = 0.1254 to 0.1784). This finding was confirmed in both urban (*C*_*n*_ = 0.1754; 95% CI = 0.1408 to 0.2101) and rural (*C*_*n*_ = 0.1191; 95% CI = 0.0779 to 0.1603) households. As indicated in Fig. 2, the concentration curve for MH use for the whole sample lies below the 45-degree line, suggesting that MH use over the past month was more concentrated among richer households.

**Table 3:** The normalized concentration indices for MH use for whole sample, and rural and urban areas in Iran

Sample	n	The ***C***_***n***_	95% Confidence interval
Urban	20,313	0.1754	0.1408 to 0.2101
Rural	18,546	0.1191	0.0779 to 0.1603
Total	38,859	0.1519	0.1254 to 0.1784

Cn is the normalized relative concentration index

**Fig. 3:**
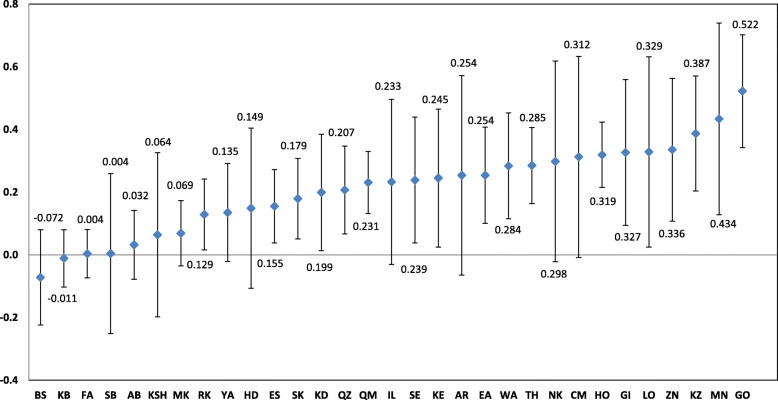
The normalized concentration index (Cn) for use of Herbal medicine across Iranian provinces, 2018

Figure 3 shows the *C*_*n*_ in MH use across provinces in Iran. While MH use was found to be concentrated among the poor households in the Bushehr and Kohgiluyeh Buyer Ahmad provinces, a higher concentration of MH usage was found among the rich households in the other 29 provinces.

### Determinants of socioeconomic inequalities in MH use

The results of the decomposition analysis on socioeconomic inequalities in MH use among Iranian households are reported in Table 4. Our findings indicate that the probability of MH use was higher in rural households (see the second column of Table 4; marginal effect). We also found that the probability of MH usage among the wealthiest households was 1.7% higher as compared to the poorest households. Based on the results of *C*_*x*_ of explanatory variables, female-headed households, illiterate-headed households, and older household heads were relatively poor. In addition, households living in rural areas were relatively rich as compared with that’s ones.

**Table 4:** Decomposition of socioeconomic inequalities in MH use among Iranian households

	Marginal effect	Elasticity	*C*_*x*_	Contribution to the *C*_*n*_		
				Contribution	%	Summed%
Demographic variables						
*Sex of household head*						
Male (ref.)						
Female	0.0006	0.0016	−0.3057	− 0.0005	− 0.3	− 0.3
*Age of household head*						
15–45 (ref.)						
46–65	0.0002	0.0015	0.0855	0.0001	0.1	
66 and above	0.0016	0.0064	−0.1626	−0.0011	− 0.7	− 0.6
Socioeconomic variables						
*Education status of household head*						
Illiterate (ref.)						
Primary school	0.0052	0.0141	−0.0260	−0.0004	−0.3	
Secondary school	0.0024	0.0488	0.0458	0.0023	1.5	
High school	0.0128	0.0492	0.1699	0.0088	5.8	
Academic degree	0.0117	0.0000	0.2844	0.0000	0.0	7.1
*Wealth index of households*						
Poorest (ref.)						
Poor	0.0092	0.0393	−0.4021	−0.0166	−10.9	
Middle	0.0121	0.0515	−0.0018	−0.0001	− 0.1	
Rich	0.0160	0.0678	0.3987	0.0284	18.7	
Richest	0.0172	0.0733	0.7996	0.0615	40.5	48.2
Ecological variables						
*Geographical area*						
Urban (ref.)						
Rural	−0.0103	−0.1042	0.0943	−0.0103	−6.8	−6.7
*Province category based on HDI*						
Low (ref.)						
Middle	0.0082	0.0570	−0.0296	−0.0018	−1.2	
High	0.0290	0.1917	0.1654	0.0333	21.9	20.7
Sum				**0.1037**		**68.3**
Residuals				**0.0482**		**31.7**
The *C*_*n*_				**0.1519**		**100.0**

The fifth and sixth columns of Table 4 show the absolute and percentage contribution of explanatory variables to the socioeconomic-related inequalities in MH use. According to the results of the decomposition analysis, the main contributing factors to the concentration of MH usage among the socioeconomically advantaged households in Iran were the economic status of households (48.2%), development status of the province (20.7%), and educational attainment of the household head (7.1%).

Of the total socioeconomic-related inequalities in MH use, 68.3% was explained by the factors included in the analysis. The remaining 31.7% of the observed inequalities in MH is explained by other variables, such as ethnicity and health condition, that are not included in the model.

## Discussion

The use of medicinal herbs as a self-care strategy is a major part of health systems in a majority of countries. In some cases, it is also the only source of care for many people. MH use is increasing, and its role in individual health is well-established [3]. While there is some evidence on the prevalence of MH use and its key determinants in Iran, there is no information on socioeconomic-related inequalities in MH use at the national and sub-national levels. This study was designed to measure the extent of socioeconomic inequalities in MH use across Iran’s provinces. Furthermore, the observed socioeconomic inequalities in MH use were decomposed to find the important determinates of these inequalities among Iranian households.

According to the finding of this study, the overall prevalence of MH use over the past month among the households was 4.7% (95% CI: 4.5–4.9%). The magnitude of MH use in this study is lower than studies done in other countries [1, 6, 9]. Deanne and colleagues reported that 63.5% of AIDS patients in Uganda had used MH after HIV diagnosis [22]. Results of a systematic review study in sub-Saharan Africa show that MH was the most common type of CAM that is used by patients, the prevalence of which was 25–65% [11]. These differences in the MH use might be explained by study population characteristics and the cultural and developmental differences among countries. In the present study, MH use was studied among the general population, but previous studies have mainly focused on populations with specific health conditions such as hypertension, diabetics, and HIV [6, 11, 18, 20–22].

The prevalence of MH had a considerable diversity across provinces, varying from 0.2 to 16.5%. This variation might be due to the socio-demographic composition of communities, climate diversity, cultural identities, and differences in beliefs. While the prevalence of MH in the Lorestan province was lowest, the use of it in the Fars province was higher than in other provinces. Climatic variations in the Fars province creates an opportunity for an extensive range of plants from diverse families to grow, and this determinant has had the most for the province to turn to the largest medicinal plants of the country [15]. The results showed that MH use was more concentrated among individuals with high SES. This finding can be explained by the fact that higher SES individuals can afford to buy medicinal herbs due to their higher ability to pay. This observation is consistent with previous studies, in which affordability is one of the most important factors for MH usage [17–19, 31, 32]. A study on the use of herbal medicine in Taiwan indicated that CHM use among pregnant women with a higher level of education and larger income had greater rates of CHM usage [33]. Additionally, a higher concentration in MH use in higher SES households is also found in both urban and rural regions. Our decomposition analysis revealed that the SES of households and residing province are the main contributing factors to the concentration of MH among the more advantaged households in Iran.

Although MH was used by people of all incomes in both urban and rural settings, our study indicated that the prevalence of MH was higher in female-headed households, households with older age, illiterate-headed households, and urban households. Female-headed households were noted to use MH more frequently than male-headed households. Consistent with our results, Geissler and colleagues found that with increasing age, females relied more heavily on MH than males [34]. Also, the lower educational level was found to be associated with a higher probability of MH use in Nigeria [35].

Our analysis indicated that there is a positive association between the age of household head and MH use, which is consistent with studies conducted by Aboyade et al. [36] in South Africa, Ching et al. [37] in Malaysia, and Chung-Hsuen Cohen et al. in the US [38]. The prevalence of MH usage was higher among residents in urban than in rural areas. This difference in herbal use can be potentially explained by the ease of access to drug shops and pharmacies that sell herbal medicine in urban areas. Evidence from previous studies shows that pharmacy and drug-shop can facilitate the use of alternative and complementary medicine [39]. Additionally, previous studies showed that urban dwellers were more likely to seek healthcare than their rural residents [36, 40].

Several limitations of this must be acknowledged. First, the cross-sectional design did not allow us to extract causal conclusions between MH usage and other factors. Second, the study utilized self-reported measures; thus there is a potential for recall bias in reporting the use of any herbal medicine over the past month. Despite these limitations inherent to survey data, findings extend knowledge of inequalities in MH use because we use data from nationally representative surveys.

## Conclusions

This study demonstrated that MH use is more concentrated among households with high SES in Iran and its provinces. Urban households, households with high socioeconomic status, female-headed households, and households headed by older people were more likely to use medicinal herbs. This finding might be in contrast with the popular belief that wealthy and socioeconomically advantaged populations, compared to low SES groups, tend to seek disproportionately more modern medical treatments and medications than MH. Understanding the factors affecting MH use, socioeconomic inequality in the use of MH and its determinants provide an opportunity for health policymakers to design effective evidence-based interventions among providers and consumers of MH.

## Data Availability

The data used in the study was extracted from the Household Income and Expenditure Surveys (HIESs) collected by the Iranian Statistical Center [41]. The HIES are can be accessed from https://www.amar.org.ir/english/Statistics-by-Topic/Household-Expenditure-and-Income#2220530-releases.

## Abbreviations

MH: Medicinal herbs
HIES: Household Income and Expenditure Survey
PCA: Principal component analysis
C_n_: Normalized concentration index
SES: Socioeconomic status
